# Meiotic Cohesin and Variants Associated With Human Reproductive Aging and Disease

**DOI:** 10.3389/fcell.2021.710033

**Published:** 2021-08-02

**Authors:** Rachel Beverley, Meredith L. Snook, Miguel Angel Brieño-Enríquez

**Affiliations:** ^1^Division of Reproductive Endocrinology and Infertility, Department of Obstetrics, Gynecology, and Reproductive Sciences, University of Pittsburgh, Pittsburgh, PA, United States; ^2^Magee-Womens Research Institute, Department of Obstetrics, Gynecology, and Reproductive Sciences, University of Pittsburgh, Pittsburgh, PA, United States

**Keywords:** cohesin, aging, reproduction, human, primary ovarian insufficiency, meiosis

## Abstract

Successful human reproduction relies on the well-orchestrated development of competent gametes through the process of meiosis. The loading of cohesin, a multi-protein complex, is a key event in the initiation of mammalian meiosis. Establishment of sister chromatid cohesion via cohesin rings is essential for ensuring homologous recombination-mediated DNA repair and future proper chromosome segregation. Cohesin proteins loaded during female fetal life are not replenished over time, and therefore are a potential etiology of age-related aneuploidy in oocytes resulting in decreased fecundity and increased infertility and miscarriage rates with advancing maternal age. Herein, we provide a brief overview of meiotic cohesin and summarize the human genetic studies which have identified genetic variants of cohesin proteins and the associated reproductive phenotypes including primary ovarian insufficiency, trisomy in offspring, and non-obstructive azoospermia. The association of cohesion defects with cancer predisposition and potential impact on aging are also described. Expansion of genetic testing within clinical medicine, with a focus on cohesin protein-related genes, may provide additional insight to previously unknown etiologies of disorders contributing to gamete exhaustion in females, and infertility and reproductive aging in both men and women.

## Introduction

Infertility, a disease defined by the failure to achieve a successful pregnancy after 12 or more months of regular, unprotected intercourse or due to an impairment of a person’s capacity to reproduce (Practice Committee of the American Society for Reproductive Medicine, 2020), is estimated to affect approximately 15% of the reproductive age population. A woman’s age is the single most important factor determining her ability to conceive as the quantity and quality of oocytes decrease progressively with advancing age (American College of Obstetricians and Gynecologists Committee on Gynecologic Practice and Practice Committee, 2014; Steiner and Jukic, 2016). The number of primordial germ cells, or oogonia, peaks at approximately 6–7 million during mid-gestation, followed by progressive atresia with approximately 1–2 million oocytes present at birth (Motta et al., 1997; Pereda et al., 2006; Mamsen et al., 2012). By puberty, there is estimated to be 300,000–500,000 oocytes remaining, and approximately 25,000 at age 37 years (Forabosco and Sforza, 2007; Albamonte et al., 2019). Menopause occurs when the number of remaining oocytes falls below a critical threshold of about 1000, regardless of age (American College of Obstetricians and Gynecologists Committee on Gynecologic Practice and Practice Committee, 2014).

Intentional delays in childbearing until later maternal age has been an increasing phenomenon over several decades. In the United States, between 1970 and 2006, the proportion of first births to women age 35 or over increased nearly eight times (Matthews and Hamilton, 2014). This trend has been attributed to multiple factors including women’s educational and professional pursuits as well as increasing availability of contraception (Simoni et al., 2017; Kavanaugh et al., 2021). Additionally, surveys of older women have demonstrated misperceptions about the magnitude of fertility decline with age. Reasons for this include recollections of messaging about pregnancy prevention starting in adolescence, healthy lifestyle and family history of normal fertility, as well as incorrect information from media reports of pregnancies in older celebrity women (Mac Dougall et al., 2013).

With later maternal age, women experience a gradual decrease in fecundity and increased rates of miscarriage (American College of Obstetricians and Gynecologists Committee on Gynecologic Practice and Practice Committee, 2014). These poor outcomes with advancing age reflect a decline in oocyte quality which can be associated with no or abnormal fertilization, no implantation, or aneuploidy (notably trisomy) of conceived embryos (Balasch and Gratacos, 2012). This manifests clinically as a higher incidence of infertility, early pregnancy loss, or developmental defects in ongoing pregnancies with increasing maternal age.

Throughout a woman’s reproductive life, successful meiosis requires homologous recombination-mediated DNA repair and proper segregation of chromosomes and sister chromatids to yield haploid oocytes. Sister chromatid cohesion, mediated by cohesin rings which tether the two sister chromatids of replicated chromosomes, is essential to ensure that these processes are carried out correctly. Loss or weakening of chromosome cohesion with advancing maternal age has been proposed as a leading cause of age-related aneuploidy in oocytes (Chiang et al., 2010, 2011; Jessberger, 2012; Herbert et al., 2015; Gruhn et al., 2019). Therefore, it is likely that deterioration of the complex of proteins involved in chromosome cohesion, referred to as cohesin proteins, or genetic variants affecting these proteins are potential drivers of age-related infertility and aneuploidy. This review provides an overview of meiotic cohesin and summarizes the human genetic studies that have identified genetic variants of cohesin proteins and the associated reproductive phenotypes.

## Meiosis and Cohesin

Understanding the etiology of aneuploidy with advancing maternal age requires an understanding of female mammalian meiosis. Mammalian meiosis is a specialized form of cell division characterized by a single round of DNA replication, followed by two rounds of chromosome segregation resulting in haploid gametes. Segregation of homologous chromosomes occurs during meiosis I (MI) and segregation of sister chromatids occurs during meiosis II (MII) (Zickler and Kleckner, 2015; Bolcun-Filas and Handel, 2018). Female mammalian meiosis commences during fetal life. Chromosomes within oocytes undergo replication and subsequently enter meiotic prophase I, and arrest in this stage for decades until future ovulation or atresia.

**FIGURE 1:**
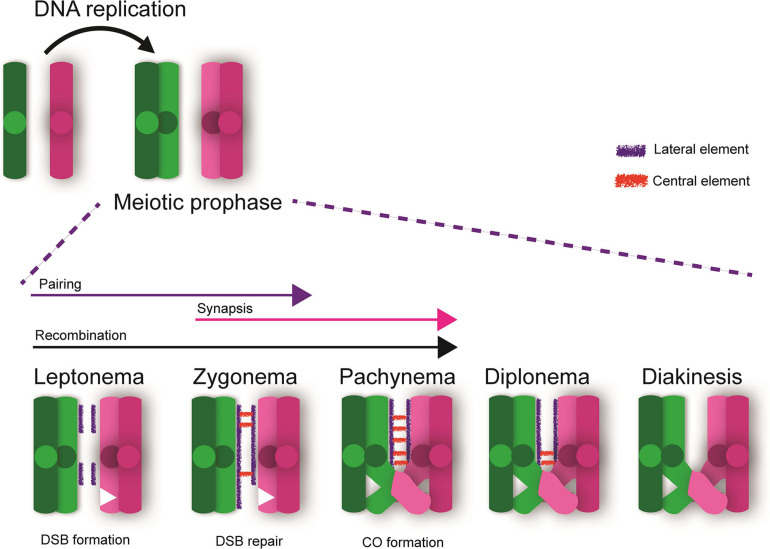
Meiotic Prophase I. Following DNA replication, meiotic prophase initiates. DNA double strand breaks (DSBs) are created during leptonema and the chromosomes initiate the processes of pairing and recombination. These processes progress into zygonema with DSB repair mechanisms ensuring proper homologous recombination. By pachynema, the chromosomes are fully synapsed and sites of crossovers (CO) are apparent. Sites of CO tether the homologous chromosomes together until anaphase I, allowing for proper segregation of homologous chromosomes and reducing the risk of aneuploidy in future gametes. Diplonema is marked by breakdown of the central element of the synaptonemal complex and chromosomes begin to repel each other. By diakinesis, homologous chromosomes are only tethered at the sites of crossovers and sister chromatids at the centromeres.

Meiotic prophase is the first and longest stage of mammalian meiosis in which the homologous chromosomes must pair, synapse, and undergo meiotic recombination to generate a crossover event. Meiotic prophase I include unique processes which are distinct from mitotic prophase. Following pre-meiotic DNA replication, meiotic prophase I initiates and progresses through various stages including leptonema, zygonema, pachynema, diplonema, and diakinesis (Zickler and Kleckner, 1999, 2015; Figure 1). Pairing-synapsis and recombination are hallmarks of prophase I, and are both essential for ensuring homolog interactions leading to the formation of at least one crossover event per chromosome pair. In fact, the number of crossovers and their location is critical for ensuring appropriate disjunction at metaphase I and for maintaining genomic stability. Pairing of homologous chromosomes occurs through the formation of a proteinaceous structure called the synaptonemal complex that forms between homologous chromosomes, while crossover recombination occurs at the DNA level, between two non-sister chromatids (Svetlanov and Cohen, 2004; Holloway and Cohen, 2015; Gray and Cohen, 2016). During leptonema, synaptonemal complex proteins (e.g., SYCP2/3) begin to form a proteinaceous scaffold (axial elements) along each homologous chromosome (Dietrich et al., 1992; Page and Hawley, 2004; Fraune et al., 2012; Cahoon et al., 2019). During zygonema, central element proteins (e.g., SYCP1, TEX12), begin to localize between the lateral elements allowing for continued pairing and synapsis between homologous chromosomes, essentially providing a proteinaceous structure physically tethering them by the end of this stage. At pachynema, lateral elements are completely formed, and the homologous chromosomes are completely synapsed (Zickler and Kleckner, 2015; Cahoon and Hawley, 2016; Gao and Colaiacovo, 2018). During diplonema, the central element of the synaptonemal complex breaks down and the chromosomes begin to repel each other. By diakinesis, homologous chromosomes are only tethered at the sites of crossovers and sister chromatids at sites of centromeres (Figure 1).

Recombination during prophase I occurs at the DNA level, between two non-sister chromatids (Svetlanov and Cohen, 2004; Holloway and Cohen, 2015; Gray and Cohen, 2016). Programmed double strand breaks (DSBs) are generated by the protein SPO11 throughout the genome in a very controlled and specific fashion. After creating hundreds of DSBs, they are repaired either as crossovers (COs) or non-crossovers (NCO) (Keeney and Kleckner, 1995; Keeney, 2008; Massy, 2016; Robert et al., 2016) or can undergo inter-sister repair as well (Garcia-Muse et al., 2019; Almanzar et al., 2021; Toraason et al., 2021). Generation of COs of homologs is required for proper segregation of homologous chromosomes during MI. The resulting bivalent chromosomes are linked at chiasmata which correspond to sites of crossovers. COs are stabilized by cohesin rings encompassing the sister chromatids distal to these sites on the chromosome arms (Petronczki et al., 2003). Full literature reviews about these processes have been described (Handel and Schimenti, 2010; Hunter, 2015; Gray and Cohen, 2016; Pereira et al., 2020).

**FIGURE 2:**
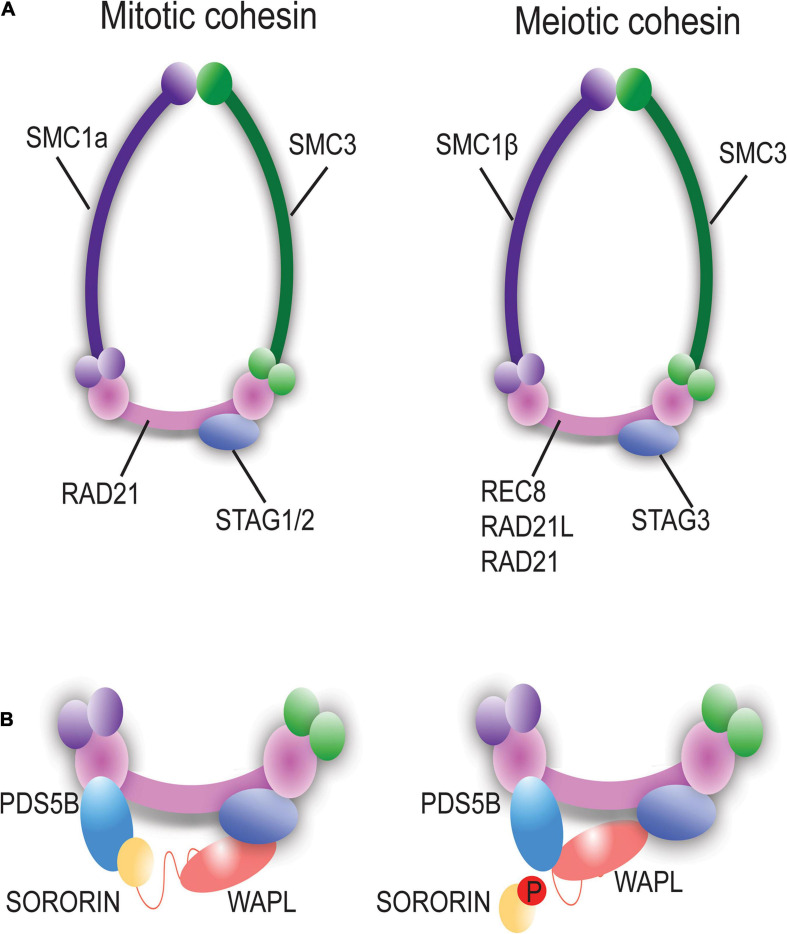
Mitotic vs. Meiotic Cohesin ring components and associated proteins. **(A)** Schematic representation of mitotic and meiotic cohesin complexes. The meiosis-specific cohesin ring proteins are SMC1β, RAD21L, REC8, and STAG3. **(B)** PDS5B, WAPL, and Sororin are associated with the cohesin complex and regulate the dynamic interaction of cohesin with chromatin. Phosphorylation of Sororin dissociates it from the cohesin ring permitting interaction of WAPL with PDS5B to open the of the cohesin ring via the prophase pathway.

Chromosome cohesion is established during pre-meiotic S phase and early prophase I via loading of cohesin rings/complexes. While mitotic and meiotic cohesin subunits are widely conserved in diverse species (Ishiguro, 2019), three types of meiotic cohesin complexes exist in mammalian cells. The subunits of STAG3, SMC1β, and SMC3 are shared in most of these complexes while the kleisin component (REC8, RAD21L, or RAD21) can vary (Herran et al., 2011; McNicoll et al., 2013; Ishiguro, 2019; Figure 2A). REC8 and RAD21L are meiosis-specific cohesin components while RAD21 is seen in both mitosis and meiosis. During meiotic prophase, REC8-containing cohesin begins to localize along the chromosomes prior to and during replication of DNA. Alternatively, RAD21L-containing cohesin rings are localized on chromosomes during leptonema and zygonema stages of prophase I and dissociates following late pachynema. The early loading of cohesin during meiosis provides a cohesin axis which serves as a structural core for chromosome organization during meiosis (Ishiguro, 2019). Thus, the cohesin complex is essential for maintaining sister chromatid cohesion and for ensuring correct chromosome segregation (Nasmyth and Haering, 2009). While mitotic and meiotic cohesion events are largely conserved, there are specific differences that exist, specifically, the unique and sequential chromosome segregation profiles during meiosis.

**FIGURE 3:**
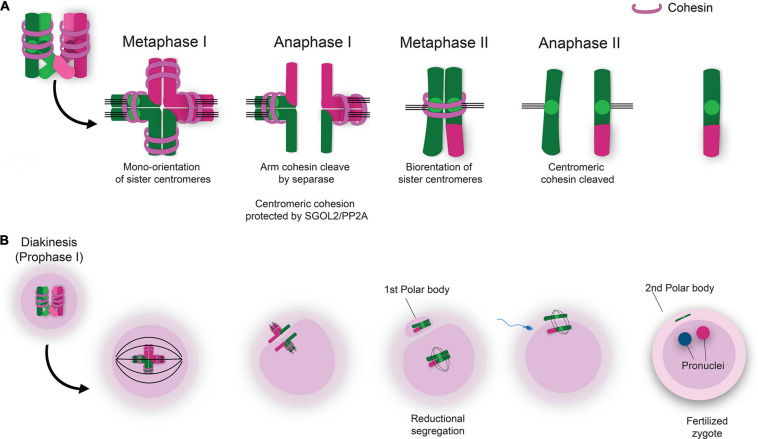
Sequential removal of cohesin ensures proper chromosome segregation and completion of meiosis. **(A)** Removal of cohesin rings from the chromosome arms during MI permits segregation of homologous chromosomes during anaphase I. Cleavage of centromeric cohesin during MII allows for segregation of sister chromatids and formation of haploid gametes. **(B)** The LH surge induces the transition of the oocyte from meiotic prophase I to complete the first meiotic division of homologous chromosomes resulting the extrusion of the first polar body and arrest at metaphase II. Fertilization is the subsequent trigger for the second meiotic division with segregation of sister chromatids and extrusion of the second polar body.

The most well-defined mechanism of cohesin removal during meiosis involves Separase-induced proteolytic cleavage (Verni et al., 2000; Waizenegger et al., 2000; Losada et al., 2002; Buheitel and Stemmann, 2013; Wassmann, 2013; Haarhuis et al., 2014; Hirano, 2015). During meiosis, cleavage of REC8 by Separase is initially restricted to the chromosome arms, and it is not until anaphase II that centromeric REC8 cleavage is initiated (Buonomo et al., 2000; Kitajima et al., 2003; Kudo et al., 2009; Ishiguro et al., 2010; Katis et al., 2010; Figure 3A). Centromeric cohesin is essential to maintain sister chromatid cohesion until the second meiotic division. These functions of cohesin ensure future proper chromosome segregation upon resumption of meiosis in sexually mature females at the time of ovulation and later during fertilization. The cohesin rings at the centromeric region are protected by the interaction of Shugoshin 2 (SGO2) and protein phosphatase 2A (PP2A) which keeps REC8 in a hypo-phosphorylated state (Kitajima et al., 2006; Riedel et al., 2006). Maintenance of centromeric cohesin rings during MI protects from premature segregation of sister chromatids (PSSC). During MII, dissociation of SGO2/PP2A at the centromeres results in phosphorylation of REC8, thus allowing the action of Separase to cleave centromeric cohesin rings which permits segregation of sister chromatids (Lee et al., 2008; Xu et al., 2009; Figure 3A).

Cohesin removal also depends on a non-proteolytic mechanism known as the prophase pathway, which precedes the action of Separase. The prophase pathway is orchestrated by an interaction between PDS5B, Sororin and wings apart-like (WAPL) protein (Verni et al., 2000; Nishiyama et al., 2013; Tedeschi et al., 2013). WAPL facilitates unloading of cohesin during prophase through an antagonistic mechanism mediated by competition between WAPL and Sororin for binding to PDS5B (Figure 2B; Kitajima et al., 2003; Kudo et al., 2009; Shintomi and Hirano, 2009; Ishiguro et al., 2010; Katis et al., 2010; Nishiyama et al., 2010; Carretero et al., 2013). In meiosis, this pathway has been described in yeast, worms, and mice (Brieno-Enriquez et al., 2016; Crawley et al., 2016; Wolf et al., 2018; Challa et al., 2019).

The proper step-wise removal of the cohesin rings is essential to ensure correct chromosome segregation during MI and MII. In sexually mature females, follicle-stimulating hormone (FSH) promotes growth of a dominant follicle in the ovary. Proliferation of granulosa cells surrounding the follicle leads to estrogen production which ultimately triggers the pre-ovulatory luteinizing hormone (LH) surge (McGee and Hsueh, 2000; Edson et al., 2009; Grive, 2020). The LH surge induces a decline in oocyte cAMP levels which then activates cyclin-dependent kinase 1 (CDK1) driving the transition of the oocyte out of prophase I arrest (Mehlmann et al., 2002; Mehlmann, 2005; Norris et al., 2010; Li et al., 2019). Removal of cohesin from the chromosome arms leads to the resolution of chiasmata and allows homologous chromosomes to segregate during anaphase I. If the first chromosome segregation commences correctly, the daughter cell receives a complete set of chromatids and the other set of chromatids are encompassed in the first polar body. Sister chromatids are maintained together via cohesin rings located at the centromere region. Mammalian oocyte meiosis then arrests at metaphase II until fertilization, as this event is the trigger for resumption and completion of MII. Resolution of centromeric cohesin rings during MII then allows for the segregation of sister chromatids. These events culminate in the formation of the haploid gamete and the second polar body (Figure 3B).

## Cohesin and Aging

Cohesin established in females during fetal life is not replenished over time during postnatal development. Oocyte cohesin expression is restricted to pre- and early meiotic prophase stages and allows for complete fertility and prevents aneuploidy (Revenkova et al., 2010). Additionally, mouse models have shown that no or very little cohesin loading occurs postnatally (Burkhardt et al., 2016). Therefore, it appears that the cohesin established during fetal life provides chromosome cohesion throughout the reproductive lifespan and the loss or weakening of chromosome cohesin may underpin the issue of declining oocyte quality and aneuploidy of aging females. Specifically, cohesin loss appears to occur while oocytes age during the time they are arrested in prophase I (Lister et al., 2010). Long-lived mouse strains as well as humans show an increased interkinetochore distance (IKD) with increasing female age (Chiang et al., 2010; Lister et al., 2010; Gruhn et al., 2019) which is thought to be a surrogate marker for loss of centromeric cohesion. Studies in human females have demonstrated that PSSC is the most common segregation error leading to aneuploidy (Pellestor et al., 2003; Garcia-Cruz et al., 2010; Ottolini et al., 2015; Gruhn et al., 2019). Therefore, weakening of centromeric cohesion is likely a major contributor to aneuploidy seen in women of advanced age. Additionally, studies of human oocytes and embryos have found higher rates of recombination and therefore sites of crossovers in euploid as opposed to aneuploid oocytes (Ottolini et al., 2015; Wang et al., 2017; Gruhn et al., 2019; Hassold et al., 2021). Given the presence of cohesin rings along the chromosome arms adjacent to the sites of crossovers, this data provides another link for the lack or loss of cohesin being a driver of aneuploidy in older women.

## Cohesin Defects and Female Reproductive Phenotypes

### Primary Ovarian Insufficiency

Primary ovarian insufficiency (POI) is defined by oligomenorrhea or amenorrhea prior to age 40 in conjunction with elevated serum FSH levels in the menopausal range, as defined by the reporting laboratory, drawn on two separate occasions at least 1 month apart (Nelson, 2009). Affecting approximately 1% of reproductive-aged women, POI is a heterogeneous disorder with a spectrum of etiologies including cytogenetic abnormalities, autoimmune factors, or various genetic causes. Additionally, POI may result from iatrogenic factors such as gonadotoxic cancer treatments (Nelson, 2009; Kort et al., 2014; Anderson et al., 2015; Hoekman et al., 2018) or ovarian surgeries. More recent advancements in clinical genetics have uncovered significant genetic contributions to POI, though the etiology in most spontaneous cases remains to be elucidated. Additionally, POI exhibits a spectrum of patient presentations including young girls who present with primary amenorrhea already qualifying for the diagnosis, compared to women in their thirties with subfertility/infertility and/or diminished ovarian reserve who subsequently go on to be formally diagnosed with POI once criteria are met.

Recent studies of families with POI from various ethnic backgrounds have identified several variants in the gene encoding the meiosis-specific cohesin protein STAG3 as a potential etiology for their diagnosis (Table 1; Caburet et al., 2014; Le Quesne Stabej et al., 2016; Colombo et al., 2017; He et al., 2018; Franca et al., 2019; Heddar et al., 2019). Interestingly, in all studies which have identified variants in *STAG3*, the index patient case(s) have presented with primary amenorrhea, elevated FSH levels in menopausal range, and streak gonads on ultrasound. These findings are consistent with early gonadal dysgenesis in young girls with otherwise normal 46XX karyotypes.

**TABLE 1:** Human Cohesin Variants and Reproductive Phenotypes.

Gene	Location	Case(s)	Ethnicity	Reproductive Phenotype	Sequence Variation	Amino Acid Change	Mechanism	References
*STAG3*	Exons 8–34 omitted	4 affected sisters	Palestinian	PA^a^, POI^b^	1bp deletion c.968delC	p.Phe187fs*7	Frameshift mutation and premature stop codon	Caburet et al., 2014
*STAG3*	Exons 19–32 omitted	2 affected sisters	Lebanese	PA, POI	2bp duplication c.1947_48dupCT	p.Tyr650Serfs*22	Frameshift mutation and premature stop codon	Le Quesne Stabej et al., 2016
*STAG3*	Skipped exon 15, out-of-frame fusion of exon 14–16	2 affected sisters	Han Chinese	PA, POI	Homozygous donor splice site mutation c.1573 + 5G > A	p.Leu490Thrfs*10	Aberrant splicing results in frameshift mutation and premature stop condon	He et al., 2018
*STAG3*	Exon 7	2 affected sisters	Asian	PA, POI	c.677C > G	p.Ser227*	Missense mutation and premature stop codon	Colombo et al., 2017
*STAG3*		1 affected woman	Brazilian	PA, POI	1bp duplication c.291dupC Nucleotide substitution c.1950C > A	p.Asn98GInfs*2 p.Tyr650*	Frameshift mutation and premature stop codon Change Tyr to Premature stop codon	Franca et al., 2019
*STAG3*	Truncating mutation in exon 28 Exon 7 substitution	2 affected sisters	Caucasian	PA, POI	c.3052delC c.659T > G	p.Arg1018Aspfs*14 p.Leu220Arg	Frameshift mutation and premature stop codon	Heddar et al., 2019
*STAG3*	Protein lacks armadillo-type domain Exon 23	1 affected male	–	MA^c^, NOA^d^	c.1762dupG c.2394 + IG > A	p.Ala588GlyfsTer9	Frameshift insertion and premature stop codon Splicing variant	Riera-Escamilla et al., 2019
*STAG3*	Exon 13	1 affected male	German	MA, NOA	c.1262T > G c.1312C > T	p.Leu421Arg p.Arg438Ter	Missense variant changes a conserved amino acid Nonsense substitution and premature stop codon	van der Bijl et al., 2019
*STAG3*	Splicing variant in intron 2 Frameshift deletion in exon 16	1 affected male	–	MA, NOA	g.100180673del c.1645_1657del	p.His549AlafsTer9	Splicing variant Frameshift deletion leads to premature stop codon at amino acid 558	Krausz et al., 2020
*RAD21L*				Maternal non-disjunction of Chromosome 21	rs450739		Missense	Chernus et al., 2019
*RAD21L*	Exon 10 Exon 14	NOA patients vs. fertile controls	Japanese	MA, SCOS^e^, NOA	c.1268A > C c.1610G > A	p.His423Pro p.Ser537Asn	Non-synonomous substitutions	Minase et al., 2017
*RAD21L*	Removal of last 41 amino acids of the protein	1 affected male	–	MA, NOA	c.1543C > T;	p.Arg515Ter	Stopgain homozygous variant	Krausz et al., 2020
*SMC1B*		2 unrelated patients		SA^f^, POI^g^ PA, POI^h^	c.662T > C c.3530A > T	p.Ile221Thr p.Gln1177Leu		Bouilly et al., 2016
*REC8*		2 affected sisters		PA, POI^i^	c.461A > G c.899G > T	p.Gln154Arg p.Arg300Leu		Bouilly et al., 2016

*^*a*^PA: primary amenorrhea.*

*^*b*^POI: primary ovarian insufficiency.*

*^*c*^MA: meiotic arrest.*

*^*d*^NOA: non-obstructive azoospermia.*

*^*e*^SCOS: Sertoli cell-only syndrome.*

*^*f*^Secondary amenorrhea.*

*^*g*^This patient was also noted to have 2 variants in *NOBOX*.*

*^*h*^This patient was also noted to have variant in *BMP15*.*

^*i*^These sisters were also noted to have variant in *GDF9*.*indicates translation stop.

The first report of mutant cohesin leading to POI was reported by Caburet et al. (2014), in a Palestinian family with four affected sisters and one affected maternal aunt. Whole exome sequencing (WES) was performed on one affected sister and one unaffected sister which identified a deleterious 1 bp deletion (c.968delC) in *STAG3* resulting in a frameshift mutation and subsequent premature stop codon (Caburet et al., 2014). Ultimately, the four affected sisters were found to be homozygous for the mutation while unaffected family members were either heterozygous or homozygous for the non-mutant allele. These authors created a *Stag3*^–/–^ mouse model which had no overt phenotype beyond sterility in both male and female mice. Histologic analysis of ovaries from these mice revealed a lack of oocytes and ovarian follicles as well as dense stroma indicating severe and early ovarian dysgenesis. Fetal oocyte meiotic chromosome spreads also demonstrated that oocytes from the *Stag3*^–/–^ mouse model had loss of centromeric sister chromatid cohesion and axial elements did not progress beyond the leptonema stage of meiotic prophase. Additionally, findings from a different *Stag3*^–/–^ mouse model demonstrated disruption in other meiosis-specific cohesin localization to the chromosome cores which led to disruption in DNA repair processes, synapsis of chromosomes, and pericentromeric heterochromatin clustering. This culminated in disruption of centromeric cohesion during meiotic prophase I with early prophase I arrest and apoptosis of both male and female germ cells (Hopkins et al., 2014). Therefore, studies in mice support the finding that this family (and others in which *STAG3* variants have been identified) has POI on the more severe end of the spectrum with diagnosis at a young age with primary amenorrhea, elevated FSH levels, and streak gonads on ultrasound consistent with a clinical picture of gonadal dysgenesis.

The second report of a *STAG3* variant identified in a family with POI was by Le Quesne Stabej et al. (2016) in a consanguineous Lebanese family with two affected sisters who presented with primary amenorrhea and absent pubertal development. Linkage analysis and WES found a homozygous 2 bp duplication (c.1947_48dupCT) which resulted in a transcript encoding a truncated protein in the *STAG3* gene. Furthermore, Colombo et al. (2017) reported on a consanguineous family of Asian origin with two sisters affected by POI. WES identified a C to G transversion at nucleotide c.677 (c.677C > G) in *STAG3*–both affected sisters were homozygous for this mutation which resulted in a premature stop codon and thus, predicted a truncated protein (p.[Ser227^∗^]). Interestingly, the 87-year-old paternal grandmother and 48-year-old maternal aunt of the sisters were both carriers of this mutation and did not suffer from amenorrhea or infertility, though they did go through premature menopause at age 40 and 37 years old, respectively.

In addition, two sisters affected with POI from a Han Chinese family were found to have a homozygous donor splice site mutation in *STAG3* (c.1573 + 5G > A) which was predicted to result in a frameshift mutation and premature stop codon. The unaffected parents and brother in this family were heterozygous carriers for the mutation (He et al., 2018). Additionally, more recent reports have shown the impact of compound heterozygous *STAG3* variants on families with POI. Heddar et al. (2019) reported on two Caucasian sisters with non-syndromic POI. WES of the proband and unaffected mother identified two novel pathogenic variants including a 1 bp deletion in exon 28 of *STAG3* (c.305delC) which yielded a premature stop codon predicting a truncated protein. The second variant was a T to G substitution in exon 7 (c.659T > G) which led to a missense mutation. Interestingly, this variant was also identified in the unaffected mother who proceeded through menopause at age 51. Franca et al. (2019) identified two rare loss-of function variants in *STAG3* (c.291dupC and c1950C > A) leading to POI in a 21-year-old Brazilian woman. Compound heterozygosity for these variants was felt to be the mode of inheritance given the rarity of both variants and their impact on the transcript and protein; however, the affected patient was adopted and therefore parental DNA was not available to confirm this assumption.

Additional cohesin-ring components have also been implicated in human POI, specifically, SMC1β and REC8 (Bouilly et al., 2016). Bouilly et al. (2016) used multiplex sequencing technology to screen 100 patients with unexplained POI for 19 different candidate genes either known or suspected to play a role in POI pathogenesis. Four patients were identified to harbor variants in genes encoding cohesin-associated proteins. Three of these patients presented with primary amenorrhea and two of them were also siblings. These sisters were diagnosed with POI at age 14 and 17, respectively. They had inherited a *REC8* variant (c.641A > G; Q154R) from their mother and a variant in *GDF9* (c.1360C > T; R54C) from their father. Both variants were predicted to be damaging by the Polyphen2 database (prediction of functional effects of human nsSNPs), a tool which predicts the potential impact of amino acid changes on the structure and function of a protein. The other patient who presented with primary amenorrhea was diagnosed with POI at age 13 and was found to have a variant in *SMC1*β (c.3530A > T; Q1177L) as well as *BMP15* (c.13A > C; S5R), both of which were predicted to be damaging by the Polyphen2 database. An additional *SMC1*β variant (c.662T > C; I221T) was identified in one patient with secondary amenorrhea and POI diagnosed at age 22, yet she was also found to have two variants in the oocyte-specific gene, NOBOX. The findings of these studies demonstrate that variants in genes encoding cohesin-associated proteins may play a role in POI pathogenesis. Moreover, digenic and perhaps polygenic inheritance likely plays a role in the timing of onset of POI as well. Future studies of patients with POI, and their evaluation for potential etiologies, may need to consider these candidate genes, and specifically *STAG3*, given multiple affected families across various ethnic backgrounds have been identified to harbor these variants.

### Trisomies

The vast majority of meiotic errors detected in human pregnancies result from errors in female meiosis and are a leading cause of pregnancy loss (Hassold and Hunt, 2001; Nagaoka et al., 2012). Meiosis in females is prone to segregation errors such as non-disjunction of homologous chromosomes as well as PSSC resulting in aneuploidy, with trisomy being the most common aneuploidy. While all autosomal chromosomes are susceptible to missegregation (Handyside et al., 2012), most autosomal aneuploidy is not compatible with embryogenesis or implantation. Therefore, autosomal aneuploidy manifests clinically as infertility or subfertility, especially in women at advanced ages. Some pregnancies affected by trisomy can progress to later stages, but most commonly result in miscarriage. Trisomy 13, 18, or 21 can result in live births with children being affected by Patau Syndrome, Edwards Syndrome, or Down Syndrome, respectively. Of these, Trisomy 21 is the most common and the incidence has been increasing in recent decades, which is likely related to women having children later in the reproductive lifespan (Morris and Alberman, 2009; Loane et al., 2013). Given that Trisomy 21 is one of the few chromosomal aneuploidies which may survive to a live birth, studying families (parents and affected child) is a resource to further understand mechanisms of meiotic segregation errors in humans.

Regarding trisomy 21, both increased maternal age as well as altered recombination events, regardless of age, have been associated with meiotic errors (Lamb et al., 1997; Oliver et al., 2008; Ray et al., 2018). The absence of recombination or the presence of a single peri-telomeric recombination event on the long arm of chromosome 21 have been associated with errors during MI and appear to be age-independent, and thus, the more common etiology of trisomy 21 in younger women (Lamb et al., 1997; Oliver et al., 2008; Ray et al., 2018). Errors during MII are associated with increasing maternal age and a peri-centromeric recombinant event on chromosome 21 (Lamb et al., 1997; Oliver et al., 2008, 2012; Ray et al., 2018). This peri-centromeric recombination pattern may lead to a suboptimal configuration which could compromise proteins involved in centromeric cohesin, exacerbating the normal degradation of this complex with age. Alternatively, the peri-centromeric recombinant event may in fact stabilize the tetrad through MI, leading to enrichment in errors during MII in older oocytes (Oliver et al., 2008).

A recent study by Chernus et al. (2019) was undertaken to discover genetic variants which may increase the risk for maternal non-disjunction of chromosome 21 using a candidate gene approach as well as a genome-wide association study (GWAS). The study sample included 749 liveborn offspring with a free, maternally derived, trisomy 21, as well as their available biological parents (*n* = 1,437 parents). DNA samples were genotyped on the HumanOmniExpressExome-8v1-2 array. These genotypes were then used to determine (1) if the non-disjunction error was maternally derived and (2) if this occurred during MI or MII. Subgroup analyses of mothers vs. fathers, MI mothers vs. fathers, MII mothers vs. fathers, and MI mothers vs. MII mothers were performed. Given that defects in cohesin proteins may play a role in PSSC and aneuploidy, the authors chose to assess candidate genes for meiosis-specific cohesin [*SMC1*β, *REC8*, *RAD21L* (SNP rs450739), and *STAG3*] within the subgroup analyses. Of the analyzed genes involved in the meiotic cohesin complex in this GWAS, there was a statistically significant association with a missense variant in *RAD21L* in the all mothers vs. fathers (OR 0.67) and MI mothers vs. fathers (OR 0.67) subgroup analyses, suggesting variants in this gene may be a risk factor for non-disjunction, more commonly in MI. None of the other candidate genes for cohesin-associated proteins demonstrated significance.

RAD21L is a part of the cohesin complex, which is an important regulator of chromosome dynamics and structure during mitosis and meiosis. Gene disruption of *RAD21L* in male mice leads to infertility whereas in female mice there is apparent age-related infertility (Herran et al., 2011). Additionally, RAD21L has been associated with recombination in males, but less so in females (Kong et al., 2014). Therefore, disruption of *RAD21L* seems to have sexually dimorphic phenotypes–possibly playing a larger role in recombination events in males and potentially a role related to chromosome segregation in females. The *RAD21L* variant association with trisomy 21 seen by Chernus et al. (2019) was more commonly seen in maternal MI errors which would be consistent with prior studies relating errors during MI to aberrations of recombination (Lamb et al., 1997; Oliver et al., 2008; Ray et al., 2018). Interestingly, the *RAD21L* variant association was not significant for MII errors despite RAD21L being a member of the cohesin complex. We would have hypothesized seeing an association with MII errors given their association with maternal age. The authors noted, however, that their sample size was limited and they did not see any unique age-associated variants. Therefore, further studies are needed to investigate alternative genetic variants which may be associated with trisomy 21 as well as how they relate to the timing of the meiotic error and maternal age at conception.

## Cohesin Defects and Male Reproductive Phenotypes

### Non-obstructive Azoospermia

The most severe form of male factor infertility is non-obstructive azoospermia (NOA). This occurs in approximately 1% of men of reproductive age (Tournaye et al., 2017). Approximately, 14% of those patients are found to have karyotype abnormalities (Van Assche et al., 1996) while approximately 8–10% have identified Y chromosome microdeletions [azoospermia factor (AZF) deletions] (Ferlin et al., 2007). When NOA is “idiopathic” the assumption is that there is likely a genetic factor involved that is yet to be determined. In recent years, sequencing approaches in families with members affected by azoospermia or severe oligospermia have identified candidate genes using a discovery-oriented approach. This method has not yet identified a cohesin-associated variant.

An alternative approach, utilizing information known from mouse models, has recently identified *STAG3* variants in a cohort of men with azoospermia. Riera-Escamilla et al. (2019) designed a “mouse azoospermia” gene panel consisting of 175 genes and subsequently analyzed those candidate genes via next-generation sequencing in a highly selected cohort of men with idiopathic NOA (*n* = 33). This cohort included 31 unrelated men and two brothers from a consanguineous family. After sequencing data was filtered, one patient was found to harbor two variants in *STAG3* (compound heterozygote). The *STAG3* variants included a frameshift insertion which generated a premature stop gain in the 597 amino acid; this is predicted to result in a protein lacking the entire armadillo-type domain of *STAG3* which is involved in DNA and protein interactions. The other *STAG3* variant identified was a novel splicing variant located on exon 23, an indispensable site for normal splicing. The patient’s unaffected fertile brother was only a heterozygous carrier for the frameshift mutation. Upon histologic analysis of testis biopsies including immunohistochemistry, the authors concluded that meiotic entry occurred with normal frequency, however, meiosis was noted to arrest as there was evidence of DSB formation, but failure to complete chromosome pairing. Therefore, it appears that *STAG3* mutations in this patient resulted in complete bilateral meiotic arrest (MA) of spermatogenesis resulting in NOA.

In similar fashion, van der Bijl et al. (2019) took a candidate gene approach to assessing sequence variants in *STAG3* in a different cohort of men with idiopathic NOA with MA (*n* = 28). In this analysis, the full coding region of *STAG3* was sequenced directly. These authors identified two compound heterozygous variants in *STAG3* (c.1262T > G; 1312C > T) in exon 13 leading to complete bilateral MA in an otherwise healthy human male. The c.1262T > G variant was paternally inherited and is a missense variant that changes a highly conserved neutral amino acid (Leucine) to a basic amino acid (Arginine) likely affecting protein folding and post-translational modifications. This variant is predicted to be disease-causing by all utilized prediction programs. The c.1312C > T variant was maternally inherited and is a nonsense substitution which introduces a premature stop codon (p.Arg438Ter). The index patient had bilateral small testis volumes (12–13 mL) and normal testosterone levels. The most developed germ cell type in any seminiferous tubule was the primary spermatocyte and a microdissection testicular sperm extraction (microTESE) procedure did not yield any sperm from this patient. Analysis of meiotic chromosome spreads from the spermatocytes of the affected patient found that no meiotic cells progressed through the zygonema stage of meiotic prophase I, which is consistent with MA. Additionally, compound heterozygosity was confirmed by Sanger sequencing of the patient’s parents. His brother inherited the c.1262T > G variant and spontaneously conceived two children.

In another recent study by Krausz et al. (2020) performed exome sequencing on 17 men with NOA due to MA who had a negative microTESE procedure. Interestingly, a plausible genetic cause of NOA was identified in 14 of these 17 patients. One patient with complete MA and normal sized testis was found to carry two novel heterozygous loss of function variants in *STAG3*–one splicing variant and one frameshift deletion in exon 16 which led to a premature stop codon at the 558th amino acid affecting the armadillo-type domain.

These studies are the first to demonstrate *STAG3* variants negatively impact protein function and lead to human male infertility due to MA. Similar to observations in females harboring variants in *STAG3* have been shown to be associated with POI as previously described. With further evidence of the important role of cohesin-associated *STAG3* in meiotic events in both males and females, consideration can be made for analysis of *STAG3* in men and women with otherwise “idiopathic” NOA or POI, respectively.

RAD21L is another meiosis-specific cohesin protein which interacts with structural maintenance of chromosome proteins SMC3 and SMC1α/β as well as STAG3 (Gutierrez-Caballero et al., 2011; Ishiguro et al., 2011; Lee and Hirano, 2011). *RAD21L* is transcribed abundantly in testis and localized to the lateral and axial elements of the synaptonemal complex playing an essential role in homologous chromosome synapsis during meiotic prophase I. Male mice deficient in *RAD21L* are defective in homologous chromosome synapsis which in turn leads to zygonema arrest and subsequent azoospermia with male sterility in mice (Herran et al., 2011). Interestingly, age-dependent sterility is seen in female mice lacking *RAD21L* (Gutierrez-Caballero et al., 2011; Herran et al., 2011).

In the study by Krausz et al. (2020) described above, one patient with NOA was noted to have a homozygous stop gain variant in *RAD21L* (c.1543C > T; p.Arg515Ter), which resulted in the removal of the final 41 amino acids of the protein. His fertile brother was found to be a heterozygous carrier. The affected patient had bilaterally small testis and histology demonstrated complete MA. In a targeted gene approach, Minase et al. (2017) evaluated the *RAD21L* coding region in a Japanese cohort with 38 men with NOA due to MA and 140 men with NOA due to Sertoli cell-only syndrome (SCOS) and compared these men to 200 fertile controls. The *RAD21L* coding region was sequenced, and three variants were found (c.454C > A; c.1268A > C; and c.1610G > A). The distribution of two of the variants (c.1268A > C, His423Pro; and c.1610G > A, Ser537Asn) was significantly different between the NOA patients with either MA or SCOS compared with the fertile controls. The authors postulated that these amino acid substitutions may play a role in disruption of spermatogenesis in Japanese patients, however, the function of the SNPs in those positions were predicted to be benign via the PolyPhen2 database. Therefore, the relationship between these identified SNPs and a mechanistic cause of NOA has yet to be determined.

## Cancer Predisposition and Cohesin Defects

In addition to its role in sister chromatid cohesion and chromosome segregation, cohesin has been implicated in genome stability owing to its role in DNA repair and recombination. DNA damage arises continuously by various endogenous and exogenous factors and cells must possess mechanisms to repair these lesions. One mechanism is via homologous recombination (HR). In this process, BRCA2 (BRCA2 DNA repair associated) and PALB2 (Partner and Localizer of BRCA2) are key regulators and promote RAD51 activity which is essential for HR. Failure to repair DNA properly may lead to cell apoptosis or cancer (Hoeijmakers, 2001; Prakash et al., 2015). Cohesin subunits have been demonstrated to undergo various mutations in cancer (Losada, 2014; Waldman, 2020). Somatic mutations in cohesin ring components have been observed in colorectal cancer (Sasaki et al., 2021), bladder cancer (Aquila et al., 2018), and hematologic malignancies and germline mutations in cohesin or its regulators are found in cohesinopathies (Horsfield et al., 2012; Cancer Genome Atlas Research Network et al., 2013).

The cohesin-associated protein PDS5B has been shown to be a mediator of homologous recombination *in vitro*. PDS5B associates with BRCA2 in early S-phase and depletion of PDS5B compromises the localization of both RAD51 and BRCA2 to the nucleus essentially disturbing HR and leading to increased cell sensitivity to DNA damaging agents (Brough et al., 2012). Based upon these findings, Couturier et al. (2016) sought to elucidate the role of PDS5B in HR and ovarian cancer prediction. In their analysis, tumor samples were obtained from chemotherapy naïve patients undergoing surgery for ovarian cancer between 1992 and 2012. The authors found that low levels of PDS5B expression correlated with improved survival in these patients. Similarly, Brough et al. (2012) assessed the expression of PDS5B in a panel of 160 invasive breast tumors and found that levels were associated with both histological grade of the breast cancer as well as outcome in patients treated with chemotherapy. In general, lower expression of PDS5B was associated with higher grade tumors and triple negative (estrogen receptor, progesterone receptor, Her2/Neu negative) tumors. Additionally, tumors with lower expression of PDS5B had a favorable response to chemotherapy.

Regarding the meiosis-specific cohesin components, loss of heterozygosity in *STAG3* has been noted in epithelial ovarian carcinomas (Notaridou et al., 2011). In the review of the literature outlined above for women with POI and associated *STAG3* variants, there was one patient noted to have bilateral ovarian tumors diagnosed at age 19 which consisted of a gonadoblastoma on the right ovary and a complex tumor of the left ovary consisting of embryonal carcinoma, choriocarcinoma, and dysgerminoma (Caburet et al., 2014). With this finding and the knowledge that cohesin plays a role in DNA repair and genome stability, additional investigation is needed to determine if women with POI related to cohesin defects are at risk for ovarian cancer and specifically, germ cell tumors.

## Cohesin Impact on Aging and Overall Health

As described above, loss or weakening of cohesin has been implicated in reproductive aging and aneuploidy. The end result of reproductive aging is POI/menopause in females or NOA in males and human genetic variants in cohesin-protein components have been implicated in both diagnoses. It is well characterized that POI/early menopause is associated with age-related diseases such as osteoporosis, cardiovascular disease (CVD), and all-cause mortality (Qiu et al., 2013; Tao et al., 2016; Quinn and Cedars, 2018). Regarding CVD pathogenesis, estrogen is felt to be cardioprotective due to effects on vascular endothelium (Mori et al., 2000; Chandrasekar et al., 2001), hence the increase in CVD risk in patients with early menopause. An alternative hypothesis is that premenopausal CVD may have effects on ovarian microvasculature predisposing to ovarian aging (Kok et al., 2006). A recent study by Wang et al. (2021) utilizing prospective data from the Nurses Health Study II found that spontaneous abortion (SAB) was associated with a greater risk of premature death, particularly from CVD. Whether or not SAB was related to microvascular changes or genetic changes was not determined, however, the authors postulated that SAB could be an early marker for future health risk in women, though the underlying mechanisms linking SAB with premature death from CVD still need to be elucidated. Therefore, the question remains if ovarian aging leads to CVD or if general aging in the individual patient is a common underlying risk factor for both CVD and ovarian aging (Cedars, 2013). For example, Hanson et al. (2020) studied young women with poor ovarian response to stimulation in assisted reproductive technology cycles and correlated oocyte yield with DNA methylation profiles in white blood cell (WBC) samples according to the “epigenetic clock” age prediction model (Horvath, 2013, 2015). Interestingly, the authors found that poor ovarian response was associated with epigenetic age acceleration in patient WBC samples.

More recent reports have also implicated male factor infertility in diseases of aging and mortality (Glazer et al., 2017; Francesco et al., 2021). Eisenberg (2016) and Eisenberg et al. (2016) reported that men with oligospermia and azoospermia have higher risks of incident diabetes, kidney disease, and ischemic heart disease in the years following their infertility evaluation. Additionally, men with infertility are at increased risk of various malignancies including testicular, prostate, bladder, and thyroid cancers; melanoma; and hematologic malignancies (Eisenberg et al., 2015). A recent systematic review by Francesco et al. (2021), found that infertile men (as compared to fertile men) have an increased risk of death, with the highest risk being associated with azoospermia.

An underlying link connecting male and female infertility with overall health and lifespan remains uncertain due to the multifactorial pathogenesis of infertility and aging as well as heterogeneity of the literature. However, the use of infertility, specifically the most extreme versions including POI in females and NOA in males, may provide a biomarker for further counseling on genetic implications for current attempts at conception as well as the future health of patients and their offspring.

## Conclusion and Prospects

Expansion of genetic testing within clinical medicine, and specifically reproductive medicine, may offer additional insight into previously unknown etiologies of disease. Specifically focusing on cohesin protein-related genes may inform the diagnosis of disorders contributing to gamete exhaustion in females, as well as infertility and reproductive aging in both men and women. The regulation of cohesin loading and removal requires the coordination of various kinases and phosphatases and further studies are needed to gain a better understanding of these mechanisms. Given that protein kinases are an emerging class of drug targets, identification of genetic defects in cohesin-related genes or regulators may serve as potential targets for future therapeutic studies of reproductive aging.

## Author Contributions

RB, MS, and MB-E collected the references and wrote the manuscript. All authors contributed to the article and approved the submitted version.

## Conflict of Interest

The authors declare that the research was conducted in the absence of any commercial or financial relationships that could be construed as a potential conflict of interest.

## Publisher’s Note

All claims expressed in this article are solely those of the authors and do not necessarily represent those of their affiliated organizations, or those of the publisher, the editors and the reviewers. Any product that may be evaluated in this article, or claim that may be made by its manufacturer, is not guaranteed or endorsed by the publisher.
